# Allen’s Human Anatomy: Section V. Organs of Sense, Organs of Digestion, and Genito-Urinary Organs

**Published:** 1884-12

**Authors:** 


ALLEN'S HUMAN ANATOMY: *Section V. Organs of Sense, Organs of Digestion, and Genito-Urinary Organs. By* HARRISON ALLEN, M.D., *Professor of Physiology in the University of Pennsylvania. Philadelphia:* HENRY C. LEA'S SON & CO. 1883.

Dr. Allen has given origin to a work on anatomy, which surpasses the high expectations of his professional friends, and completely fills a gap in American medical literature. Section V is a beautiful book. The descriptions in the text are clear, distinct and of an intensely practical character. Errors are few in number, and usually, of such trivial importance as to deserve no attention. Mr. Herman Faber is entitled to much praise for his faithful drawings of the author's exquisite dissections.

